# Preclinical Assessment of the Combination of PSMA-Targeting Radionuclide Therapy with PARP Inhibitors for Prostate Cancer Treatment

**DOI:** 10.3390/ijms23148037

**Published:** 2022-07-21

**Authors:** Eline A. M. Ruigrok, Nicole S. Verkaik, Erik de Blois, Corrina de Ridder, Debra Stuurman, Stefan J. Roobol, Dik C. Van Gent, Marion de Jong, Wytske M. Van Weerden, Julie Nonnekens

**Affiliations:** 1 Department of Radiology and Nuclear Medicine, Erasmus MC Cancer Institute, Erasmus University Medical Center, 3000 CA Rotterdam, The Netherlands; e.ruigrok@erasmusmc.nl (E.A.M.R.); r.deblois@erasmusmc.nl (E.d.B.); c.deridder@erasmusmc.nl (C.d.R.); d.stuurman@erasmusmc.nl (D.S.); sroobol@gmail.com (S.J.R.); julie.nonnekens@gmail.com (M.d.J.); 2 Department of Experimental Urology, Erasmus University Medical Center, 3000 CA Rotterdam, The Netherlands; w.vanweerden@erasmusmc.nl; 3 Department of Molecular Genetics, Erasmus MC Cancer Institute, Erasmus University Medical Center, 3000 CA Rotterdam, The Netherlands; n.verkaik@erasmusmc.nl (N.S.V.); d.vangent@erasmusmc.nl (D.C.V.G.)

**Keywords:** prostate specific membrane antigen, targeted radionuclide therapy, radiosensitization, PARP inhibitors

## Abstract

Prostate specific membrane antigen targeted radionuclide therapy (PSMA-TRT) is a promising novel treatment for prostate cancer (PCa) patients. However, PSMA-TRT cannot be used for curative intent yet, thus additional research on how to improve the therapeutic efficacy is warranted. A potential way of achieving this, is combining TRT with poly ADP-ribosylation inhibitors (PARPi), which has shown promising results for TRT of neuroendocrine tumor cells. Currently, several clinical trials have been initiated for this combination for PCa, however so far, no evidence of synergism is available for PCa. Therefore, we evaluated the combination of PSMA-TRT with three classes of PARPi in preclinical PCa models. In vitro viability and survival assays were performed using PSMA-expressing PCa cell lines PC3-PIP and LNCaP to assess the effect of increasing concentrations of PARPi veliparib, olaparib or talazoparib in combination with PSMA-TRT compared to single PARPi treatment. Next, DNA damage analyses were performed by quantifying the number of DNA breaks by immunofluorescent stainings. Lastly, the potential of the combination treatments was studied in vivo in mice bearing PC3-PIP xenografts. Our results show that combining PSMA-TRT with PARPi did not synergistically affect the in vitro clonogenic survival or cell viability. DNA-damage analysis revealed only a significant increase in DNA breaks when combining PSMA-TRT with veliparib and not in the other combination treatments. Moreover, PSMA-TRT with PARPi treatment did not improve tumor control compared to PSMA-TRT monotherapy. Overall, the data presented do not support the assumption that combining PSMA-TRT with PARPi leads to a synergistic antitumor effect in PCa. These results underline that extensive preclinical research using various PCa models is imperative to validate the applicability of the combination strategy for PCa, as it is for other cancer types.

## 1. Introduction

Prostate specific membrane antigen targeted radionuclide therapy (PSMA-TRT) has shown promising results in castration resistant prostate cancer (CRPC) patients [1,2,3]. During PSMA-TRT, patients are intravenously treated with PSMA-specific small molecule inhibitors labeled with radionuclides with DNA-damaging capacities. Because of the overexpression of PSMA on prostate cancer (PCa) cells in the majority of the patients, PSMA-TRT accumulate in PCa tumor lesions enabling local irradiation in the PCa lesions. Lutetium-177 (β-emitter) is often the radionuclide of choice for PSMA-TRT [3,4]. Among several clinical trials, the VISION clinical phase III trial showed that treatment with a PSMA targeting PSMA-617, labeled with lutetium-177 ([^177^Lu]Lu-PSMA-617) plus standard care, significantly increased the overall survival compared to standard care alone in advanced metastatic CRPC patients, resulting in food and drug administration (FDA) approval of [^177^Lu]Lu-PSMA-617 in this patient group [4].

Unfortunately, it has been shown in this and other trials that 20–40% of PCa patients have minimal to no response to PSMA-TRT, indicating that PSMA-TRT is not effective enough yet for the majority of patients with metastatic PCa to be applied with a curative intent [5,6,7]. Thus, increasing the effectivity of PSMA-TRT (without increasing the radiotoxic effects) is of great importance. A potential way of achieving this, is combining PSMA-TRT with another therapy which induces a synergistic increased effect.

Lutetium-177 decay induces mostly DNA single-strand breaks (SSBs) in the targeted cells. SSBs that are not repaired are converted during replication into much more cytotoxic DNA double strand breaks (DSBs) [8]. Essential for the repair of SSBs is poly-[ADP-ribose]-polymerase-1 (PARP-1). When PARP-1 recognizes a damaged SSB site, it binds to the site and catalyzes poly ADP-ribosylation (PARylation), which initiates the recruitment of additional DNA repair proteins [9]. PARP-1 inhibitors (PARPi) inhibit PARP-1 by blocking PARylation and thereby inhibit the recruitment of these other DNA repair proteins. In addition, PARPi could also trap PARP-1 at the SSB which fully prevents further repair [9,10]. Various PARPi with different ‘PARP-trapping potencies’ and corresponding cytotoxic effects have been developed [11]. For example, talazoparib, a PARPi with strong PARP-trapping potency, shows the highest cytotoxic effects, while veliparib with the least potent PARP-trapping ability and relatively low effectivity in general [12,13].

Several preclinical studies in neuroendocrine tumors (NETs) have shown that combination of lutetium-177 TRT with a PARPi (often olaparib), increases cancer cell killing both in vitro and in vivo [14,15,16]. Based on these preclinical successes with NET models, clinical trials combining lutetium-177 TRT and PARPi in NET patients and in metastatic CRPC patients are currently ongoing [17]. In contrast to the preclinical studies combining TRT with PARPi in NET models, to the best of our knowledge, the phase I study for metastatic CRPC patients was initiated without available preclinical data on the potential value of this combination study in metastatic PCa. Assuming possible differences in the response to this (combination) therapy between various types of cancer and to assess the radiosensitivity potency of PARPi with different characteristics, we set out to study the cellular effects of the combination of TRT with three classes of PARPi in preclinical models of PCa.

In this preclinical study, we combined PSMA-TRT with three classes of PARPi to assess survival and viability in vitro and conducted an in vivo validation study. Unfortunately, we did not find a synergistic combinatory effect using different conditions and preclinical models. Despite this unexpected outcome these data provide valuable information on the performance of PARPi in combination with PSMA-TRT in PCa, especially in view of the ongoing clinical trials.

## 2. Results

### 2.1. PARPi Did Not Radiosensitize PCa Cells to PSMA-TRT

**Figure 1:**
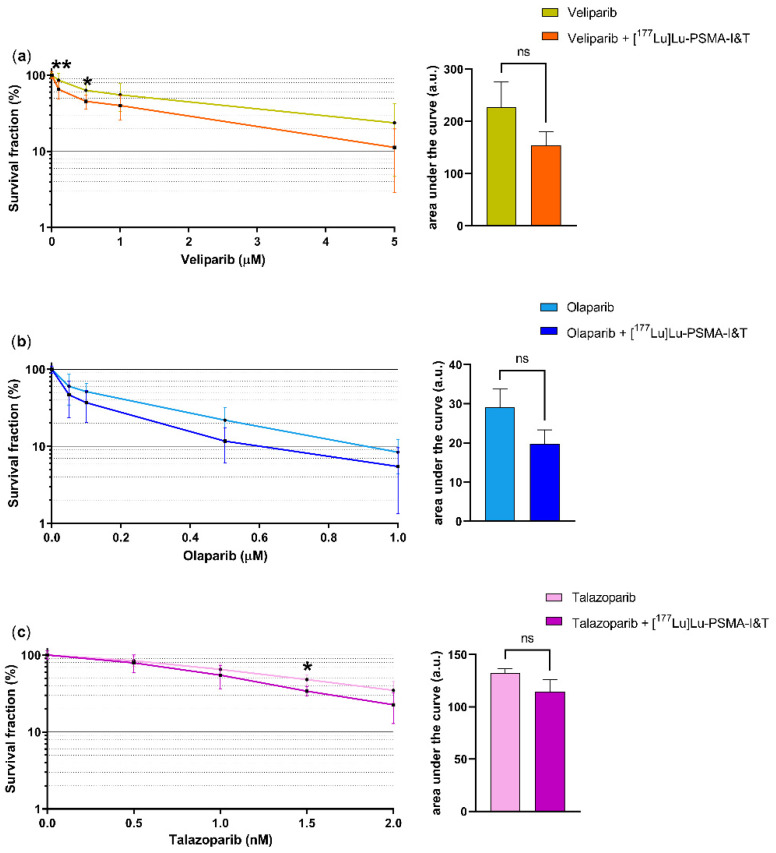
Quantification of the clonogenic survival of PC3-PIP cells treated with [^177^Lu]Lu-PSMA-I&T in combination with increasing concentrations of veliparib (**a**), olaparib (**b**) and talazoparib (**c**) compared to PARPi monotherapy and the corresponding area under the curve comparison. No PARPi conditions were used to normalize for both [^177^Lu]Lu-PSMA-I&T and the mock-treated curves. Error bars represent standard error of the mean (SEM). Asterisks indicate significance compared to PARPi monotherapy (* *p* ≤ 0.05, ** *p* ≤ 0.01).

To analyze the effect of combining PSMA-TRT ([^177^Lu]Lu-PSMA-I&T) with PARPi, clonogenic survival assays were performed using PC3-PIP cells (a PSMA transfected cell line). The quantification showed a lower, but non-significant overall survival of cells treated with [^177^Lu]Lu-PSMA-I&T combined with increasing concentrations of veliparib, olaparib or talazoparib compared to cells treated without [^177^Lu]Lu-PSMA-I&T (Figure 1). Significant decreases in survival were shown in cells treated with [^177^Lu]Lu-PSMA-I&T in combination with 0.3 µM and 0.5 µM veliparib in comparison to 0.3 µM and 0.5 µM veliparib alone. No significant decrease in survival was shown in cells treated with [^177^Lu]Lu-PSMA-I&T combined with olaparib compared to olaparib alone. Cells treated with [^177^Lu]Lu-PSMA-I&T in combination with talazoparib showed a significant difference in survival at the 1.5 nM concentration. These clonogenic survival data were validated in cell viability assays in PC3-PIP and LNCaP cells (an endogenous PSMA-expressing cell line), which showed no synergistic effect of the combination therapy (data not shown).

### 2.2. DNA Damage Induction Was Not Significantly Enhanced by the Combination Treatment

**Figure 2:**
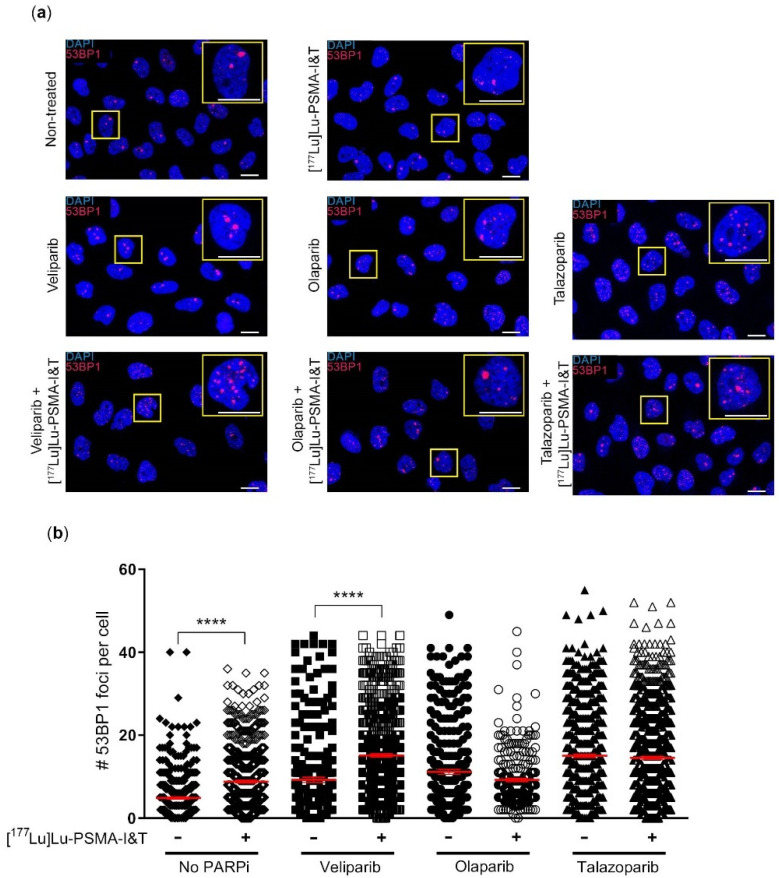
DSB foci in PC3-PIP cells treated with [^177^Lu]Lu-PSMA-I&T in combination with PARPi. DSB analysis of cells treated with [^177^Lu]Lu-PSMA-I&T with or without PARPi. (**a**) Representative images of immunofluorescent stainings. Blue: DAPI; red: 53BP1 (scale bar = 7.5 μm). (**b**) Quantification of the number of 53BP1 foci per nucleus. Error bars indicate SEM (n = 3) (**** *p* ≤ 0.0001).

In vitro survival assays showed minimal differences between cells treated with PARPi monotherapy and cells treated with PARPi in combination with [^177^Lu]Lu-PSMA-I&T. To better interpret these results and relate them to the literature of this combination treatment in NET models, the differences in DSB induction between PARPi monotherapy and combination therapy with [^177^Lu]Lu-PSMA-I&T was determined in PC3-PIP cells by analyzing the number of DSBs using 53BP1 foci formation as marker (Figure 2a).

A significant increase in the number of DSBs was seen 3 days after incubation of cells treated with [^177^Lu]Lu-PSMA-I&T compared to the untreated control. Furthermore, cells treated with 1 µM veliparib, 0.1 µM olaparib or 1.5 nM talazoparib monotherapy (the concentrations at which ~50% survival was observed during the clonogenic survival assays) also showed a significant increase in the average amount of DSBs per cell, compared to the untreated control. The combination of veliparib with [^177^Lu]Lu-PSMA-I&T led to a small, but significant increase in the average number of DSBs per cell compared to veliparib alone. Combining [^177^Lu]Lu-PSMA-I&T with olaparib or talazoparib did not lead to a significant increase in average number of DSBs compared to the PARPi monotherapy (Figure 2b).

### 2.3. PARPi Treatment Started Simultaneously with PSMA-TRT Did Not Enhance In Vivo Treatment Efficacy

Although in vitro the beneficial effects of combining PARPi treatment to PSMA-TRT were small, the results of the clonogenic survivals were showing a promising trend. Therefore, and because cell lines can behave different in vitro and in vivo, we decided to continue to test the potential of the combination treatments in vivo in the PC3-PIP xenograft model.

PC3-PIP tumor bearing mice were injected with a non-curative dose of [^177^Lu]Lu-PSMA-I&T or vehicle control in combination with daily oral dosing of one of 3 PARPi or vehicle control. All animals remained on their start weight or gained weight, except for the groups receiving a daily dose of 0.5 mg/kg talazoparib (Supplementary Materials Figure S1). The quick loss of weight of these animals in combination with signs of discomfort has led to an early discontinuation of these groups from the study.

**Figure 3:**
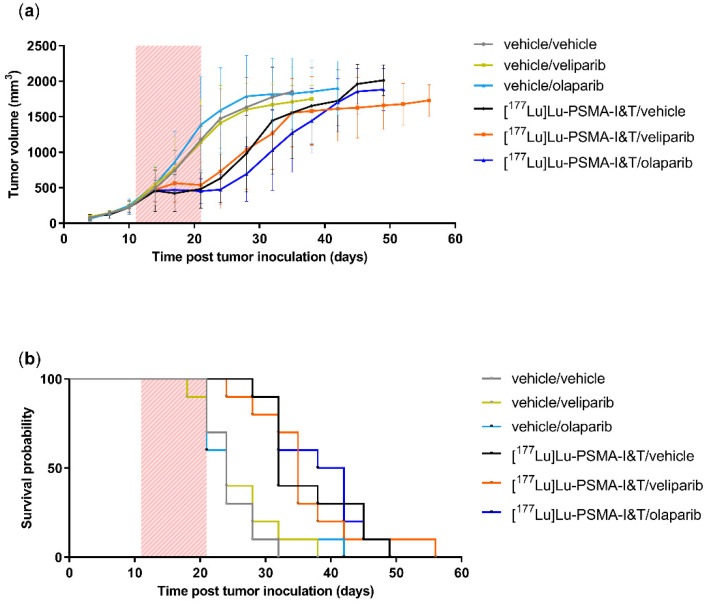
In vivo valuation of [^177^Lu]Lu-PSMA-I&T effectivity in combination with PARPi. (**a**) Average tumor volume and (**b**) survival probability. The red box indicates the treatment period with one injection of [^177^Lu]Lu-PSMA-I&T or vehicle at day 11 and subsequent daily dosing by oral gavage of the PARPi or vehicle from day 11 until day 21. Error bars indicate standard deviation. The tumor growth of each individual animal can be found in Supplementary Materials Figure S2.

A significant delay (*p* < 0.001) in tumor growth and increase of survival of approximately 10 days was observed for all animals treated with [^177^Lu]Lu-PSMA-I&T compared to the animals that received a vehicle injection. However, no differences in tumor growth and survival were seen between mice receiving [^177^Lu]Lu-PSMA-I&T/vehicle or receiving [^177^Lu]Lu-PSMA-I&T/veliparib or [^177^Lu]Lu-PSMA-I&T/olaparib when PARPi daily oral dosing was initiated simultaneously with PSMA-TRT. Importantly, no difference in tumor growth or survival was seen between the vehicle/vehicle animals and the animals receiving PARPi monotherapy (Figure 3 and Supplementary Materials Figure S2).

In order to verify if the daily oral dosing regimen of PARPi resulted in measurable levels in plasma and tumor tissue, olaparib levels were measured in animals that received vehicle/olaparib and were sacrificed within 2 h after the last olaparib administration. Olaparib could be detected in the plasma and tumor tissue of these animals, with levels up to 13 nM in plasma and 2.17 ng/mg in tumor tissue (Table 1).

## 3. Discussion

The aim of this study was to evaluate the synergistic combination of PSMA-TRT with various PARPi in preclinical models of PCa. The combination of other types of TRT with a PARPi has provided promising results in preclinical research on NET models, as PARPi inhibit the ability of the cell to repair SSBs induced by TRT, which leads to an increased amount of cytotoxic DSBs and consequently to tumor cell death [9]. Here, we determined the effect of [^177^Lu]Lu-PSMA-I&T combined with various PARPi on the viability, survival and DNA damage induction of PSMA-expressing PCa cells in vitro and in vivo.

To assess PSMA-TRT efficacy in vitro, we used two assays, the clonogenic survival assay that determines the ability of single cells to survive and form colonies, and a cell viability assay that measures the number of viable (metabolic active) cells based on the quantitation of ATP levels [18]. Clonogenic survival assays using PSMA expressing PC3-PIP cells revealed a small, but encouraging difference in survival indicating that cells treated with the PSMA-TRT and PARPi combination therapy have a lower survival compared to cells treated with PARPi monotherapy. Unfortunately, these results did not reach significant difference in the area under the curve comparison between the combination- and PSMA-TRT only treatment. Moreover, the in vitro viability assays did not reveal significant differences between the PSMA-TRT and PARPi combination therapy and the PSMA-TRT monotherapy in both examined cell lines.

In order to further identify the potential of the combination therapy, we quantified DSBs as a result of [^177^Lu]Lu-PSMA-I&T with PARPi versus the different monotherapies. In line with the survival and viability results, we found no difference in the number of DSBs in cells after [^177^Lu]Lu-PSMA-I&T in combination with olaparib or talazoparib versus olaparib or talazoparib monotherapy, respectively. We observed a significant increase in DSBs in cells treated with [^177^Lu]Lu-PSMA-I&T and veliparib compared to veliparib only. These results were in line with the results of the clonogenic survival assay, where the veliparib combination therapy showed the largest effect compared to veliparib monotherapy, although this effect did not reach statistical significance. Since veliparib is the PARPi with the weakest ‘PARP-trapping’ potency, our results, although minimal, could indicate that PCa cell survival after treatment of [^177^Lu]Lu-PSMA-I&T is not hampered by PARP-trapping [19]. If synergism could be proven in other models and/or treatment regimens, the use of veliparib for TRT combination therapy for PCa could be beneficial for clinical implementation since veliparib has a favorable low toxicity profile [20].

In the in vivo experiment, an early discontinuation of all animals that received talazoparib was necessary due to signs of toxicity. The dose of 0.5 mg/kg talazoparib was based on previous research showing no toxicity in animals (Balb/c nude mice, C.B-17 scid^−/−^ mice and NCr-nu/nu mice) receiving 0.25–0.33 mg/kg talazoparib twice a day for 5 days [14,21,22]. The observed toxicity in our NMRI (Foxn1 nu/nu) animals could be mouse strain specific or may be caused by the once daily PARPi administration leading to exceeding the maximum tolerated dose at a given time.

Our study using the PSMA-expressing PC3-PIP model did not support our hypothesis of a synergistic increased therapeutic effect of the combination of [^177^Lu]Lu-PSMA-I&T with PARPi compared to [^177^Lu]Lu-PSMA-I&T alone. Our observations are not in line with the literature that reports promising, synergistic effects when combining PARPi with other types of TRT in preclinical research. For instance, our group previously has shown that cancer cells overexpressing somatostatin receptor 2 (a commonly overexpressed receptor in NETs), synergistically sensitized to TRT when also treated with olaparib in vitro [16]. Moreover, Cullinane et al. demonstrated, next to promising in vitro results, that the combination of TRT with talazoparib led to improved anti-tumor efficacy compared to TRT alone, in the AR42J NET tumor model in vivo [14]. The cell lines used in these cited studies respond less sensitive to the PARPi monotherapy compared to the PC3-PIP cell line used in our research; AR42 cells showed a small increase in DSBs after treatment with 50 nM talazoparib, while PC3-PIP cells in our study demonstrated only a survival of 20–30% after treatment with 2 nM talazoparib. These differences in cellular response underline that sensitivity to PARPi monotherapy may strongly vary between cancer models and/or cancer types. As a consequence, the potential impact in the context of [^177^Lu]Lu-PSMA-TRT may be very different relying on additional conditions, such as optimal timing of the combination and treatment duration. Moreover, we have not yet examined the potential inherent insensitivity of these PCa models towards PARPi, which may be caused by either low levels of endogenous PARP-1 levels, the cellular ability to form PAR chains and the potential low uptake and/or rapid excretion of significant amounts of PARPi. This would be important to be investigated in future studies to assess potential cellular mechanisms of resistance.

For external beam radiotherapy (EBRT) it has been shown that olaparib radiosensitized 22Rv1 PCa cells in vitro and in vivo [23]. However, it must be noted that DNA damage induction and repair of EBR cannot be directly compared to the DNA damage induced by TRT [24]. Moreover, 22Rv1 cells display a low PSMA expression, making this model less suitable for PSMA-TRT research. To the best of our knowledge, the strategy of combining TRT with PARPi has not been demonstrated in PCa before.

Additionally, we have shown that the lack in effect in tumor control between mice treated with [^177^Lu]Lu-PSMA-I&T and olaparib or [^177^Lu]Lu-PSMA-I&T alone was not caused by insufficient levels of the PARPi to reach the tumor as olaparib levels in tumor and plasma 2 h post-injection were in line with concentrations found previously in CD1 nude mice bearing G7 glioblastoma xenografts 2 h post-injection with 50mg/kg olaparib, which was sufficient to radiosensitize these tumors [25]. This study in glioblastoma tumors also showed that 5 h post-injection, olaparib levels were nearly undetectable. This underscores that precise timing of dose schedules of both treatments may be key to obtain optimal efficacy. Therefore, future pharmacokinetic studies would be advisable to test various protocols to define the optimal time for maximal antitumor effect of the combination therapy.

In the treatment landscape for PCa patients, combination with an antiandrogen would be an interesting option to increase sensitivity. In earlier studies, we have shown that anti-androgen treatment indeed hampers DSB repair induced by external beam radiotherapy, leading to radiosensitization [26]. We did not test the combination of anti-androgens with PSMA-RLT and/or PARPi in the current study, but based on the results with external beam radiotherapy, we could expect a radiosensitization effect of PSMA-TRT by anti-androgens.

Despite the promising results with NET models and the initiated clinical trials in NET patients and in PCa patients, our current preclinical data strongly urge for further research into PSMA-TRT PCa efficacy by (1) selecting the proper preclinical models to perform the research, (2) evaluating more dedicated and optimized PSMA-TRT and PARPi therapy protocols (for example improved treatments schedules based on pharmacokinetics) and (3) defining biomarkers that could be used to predict efficacy and toxicity. The PC3-PIP cells used in this study are PSMA transfected and hence harbor relatively high levels of PSMA. It will be important in future studies to using PCa models with endogenous PSMA expression [27]. For instance, PCa models that are more closely related to the clinic, such as patient derived xenografts, could be used in future research.

The PARP inhibitor olaparib is currently approved for the treatment of PCa patients harboring a somatic or germline Breast Cancer 1 or 2 (*BRCA1*/*2)* mutation, similarly, deficiency of several other DNA damage repair (DDR) proteins has been detected in (late stage) PCa patients. These DDR aberrations may impact the response to the currently tested treatment combination, suggesting that combination therapy of PSMA-RLT and PARPi could be a promising step towards better treatment for this particular patient group.

The first clinical trial combining olaparib and PSMA-TRT is currently ongoing [17]. Our data presented here does not support the hypothesis that additional treatment with PARPi increases the clinical response of PSMA-TRT. Future preclinical research using different PCa models in vitro and in vivo is therefore imperative to validate the applicability of the combination strategy for PCa, as it is for other cancer types.

## 4. Materials and Methods

### 4.1. Reagents and Chemicals

All reagents and chemicals were purchased from Sigma-Aldrich (St. Louis, MO, USA) unless specified below.

### 4.2. Radiolabeling

For in vitro studies, lutetium-177 was obtained from IDB Holland BV an Advanced Accelerator Applications company (Baarle-Nassau, The Netherlands), and PSMA-I&T (Huayi Isotopes Co. via ATT Scintomics, Fürstenfeldbruck, Germany) was labeled with a molar activity of 40 MBq/nmol as previously described [28]. Radiolabeling for in vivo experiments, with a molar activity of 80 MBq/nmol, were conducted with Lutetium-177 n.c.a. EndolucinBeta^®^ (ITM Medical Isotopes GmbH, Munich, Germany). Quenchers (3.5 mM ascorbic acid, 3.5 mM gentisic acid, 10 mM methionine) were added to prevent radiolysis of the radiotracer. Quality control was assessed using high-pressure liquid chromatography and instant thin-layer chromatography. For all labelings, the radiochemical yield was >95% and the radiochemical purity was >90%.

### 4.3. PARP-Inhibitors

In this study PARPi veliparib, olaparib and talazoparib were used (Selleckchem, Planegg, Germany). For in vitro studies, the PARPi were dissolved in 50% dimethyl sulfoxide (DMSO) at a concentration of 1 mM before dilution to the desired concentrations in the culture medium. For the in vivo therapy study, the PARPi were solved in 4% DMSO and 30% Polyethylene glycol 300.

### 4.4. Cell Culture

In vitro experiments were performed on PSMA transfected PC3-PIP cells and on the endogenous PSMA expressing LNCaP cells (ATCC). PC3-PIP was kindly provided by prof. Anna Orlova, Uppsala University. Both cell lines were cultured in RPMI 1640, Glutamax medium (Gibco, Thermo Fisher Scientific, Waltham, MA, USA) supplemented with 10% Fetal bovine serum (Gibco), penicillin (100 units/mL) (Gibco) and streptomycin (100 μg/mL) (Gibco). Additionally, during cell culture, 10 µg/mL puromycin (Invivogen, San Diego, CA, USA) was added every other week to the PC3-PIP cells. PC3-PIP cells used during experiments were in puromycin-free medium for at least 48 h. All cells were grown at 37 °C and 5% CO_2_.

### 4.5. Clonogenic Survival Assay

PC3-PIP cells were treated for 3 h in suspension by placing 100,000 cells in 1 mL of 20 mM HEPES buffered culture medium with or without 0.3 MBq/mL (12 × 10^−9^ M) [^177^Lu]Lu-PSMA-I&T in a 2 mL safe-lock Eppendorf tube. During incubation, the tubes were placed on a rocker plate at 37 °C. After incubation, for each condition 450 cells were seeded in triplicate in 6-well plates, with or without increasing concentrations of PARPi. 7 days after [^177^Lu]Lu-PSMA-I&T incubation, the plates were washed with PBS and stained with 0.1% Coomassie brilliant blue solution (50% methanol, 7% acetic acid, 43% water, 0.1% Coomassie (Thermo Fisher Scientific, Waltham, MA, USA)) for at least 15 min at room temperature (RT). Colonies were counted using gelcounter (Oxford Optronix, Abingdon, UK). The results were normalized to 0.3 MBq/mL [^177^Lu]Lu-PSMA-I&T monotherapy treated controls. Experiments were performed as four independent experiments in triplicate. The area under the curve was calculated using GraphPad Prism software version 9.0.0 (GraphPad Software, San Diego, CA, USA).

### 4.6. DNA Damage Assay

PC3-PIP cells were treated in suspension for 3 h with or without 0.3 MBq/mL [^177^Lu]Lu-PSMA-I&T, washed 1× with PBS and seeded onto coverslips in 6-well plates, with culture medium with or without PARPi (1 µM veliparib, 0.1 µM olaparib or 1.5 nM talazoparib, the IC_50_ concentrations of the monotherapy survival assays). 3 days after [^177^Lu]Lu-PSMA-I&T incubation, the cells were washed with PBS and fixed with 2% paraformaldehyde in PBS for 15 min at RT. Next, the cells were washed once with PBS, 2 times for 10 min with PBS + 0.1% Triton X-100 and once for 30 min with PBS+ (100 mL PBS + 0.5 g BSA + 0.15 g glycine). Subsequently, the cells were incubated with the primary antibody, anti-53BP1 (1:1000, rabbit (Novus Biologicals, Abingdon, UK) in PBS+ for 90 min at RT. Hereafter the cells were washed using PBS + 0.1% Triton X-100 and incubated with the secondary antibody, goat anti-mouse Alexa Fluor 594 (1:1000 (Thermo Fisher Scientific, Waltham, MA, USA) for 60 min at RT. The cells were then washed with PBS + 0.1% Triton X-100 and mounted with Vectashield + Dapi (Vector Laboratories, Newark, CA, USA). Fluorescent imaging was performed using z-stack acquisition (63× oil lens, 2× zoom) on a SP5 confocal microscope (Leica Biosystems, Nussloch, Germany). The images were analyzed using Image J software version 1.53k (National Institutes of Health, Bethesda, MD, USA) with a custom-made macro. In short, of each image the z-stack was compressed and each cell was segmented, based on the DAPI staining, into regions of interest (ROI). Within each ROI, 53BP1-foci were segmented and counted. For each experiment, 6–8 fields of view were imaged of each condition, with 20–60 cells per image. This experiment was performed as 3 independent experiments.

### 4.7. In Vivo Therapy Study

The conducted animal experiments were approved by the Erasmus MC Animal Welfare Committee and were in accordance with the European law. NMRI (Foxn1 nu/nu) male animals (6–8 weeks, Janvier), were maintained in standard 12-h light/dark cycle with water and food ad libitum.

80 animals were subcutaneously inoculated on their right shoulder with 5 × 10^6^ PC3-PIP cells in 100 µL consisting of 33% Corning Matrigel and 66% HBSS medium (Gibco). Tumor take was 100%. For the entire duration of the experiment, body weight and tumor growth were monitored twice a week and animals were observed daily for their body condition score. Tumors were measured using a caliper, and tumor volume (TV) was calculated using the formula: π/6 (tumor length × tumor width) ^ (3/2). Animals were randomized into 8 groups based on TV and body weight. Tumors were allowed to grow to a size of (300–500 mm3), reached at day 11 after tumor inoculation, and treatments were initiated: groups 1–4 received intravenous (i.v.) injection with the vehicle control (PBS with added quenchers 3.5 mM ascorbic acid, 3.5 mM gentisic acid, 10 mM methionine), and groups 5–8 were injected with 10 MBq/125 pmol/200 µL [^177^Lu]Lu-PSMA-I&T. Immediately after TRT (or vehicle) injection, animals received PARPi or vehicle by daily oral dosing for 10 days. Groups 1 and 5 were given a vehicle control (4% DMSO, 30% PEG 300, 66% dH_2_O), groups 2 and 6 received veliparib (100 mg/kg), groups 3 and 7 olaparib (100 mg/kg) and groups 4 and 8 talazoparib (0.5 mg/kg). Concentrations PARPi and [^177^Lu]Lu-PSMA-I&T were based on literature [14,21,29,30]. The PARPi solutions were freshly prepared daily directly prior for administration. The animals were sacrificed when reaching a humane endpoint or when the tumor volume exceeded 1500 mm^3^.

### 4.8. Olaparib Measurements

To validate if the PARPi reached the tumor tissue, olaparib levels were measured in tumor and plasma of 5 animals of the vehicle/olaparib group. Four out of five animals were sacrificed approximately 2 h after the last olaparib administration. Tumor and plasma of a mouse that was sacrificed 7 days after the final olaparib treatment was included to serve as control for the olaparib analysis.

Quantitation of olaparib was performed with a fully validated ultra-performance liquid chromatography (UPLC) method with a lower limit of quantitation of 50.0 ng/mL (115 nM) olaparib in plasma. Briefly, an aliquot of 100 μL of internal standard working solution (100 ng/mL dasatinib-d8 in acetonitrile) was added to 25.0 μL of plasma sample in 1.8-mL safe-lock reaction vials and vigorously mixed for 5 s. After centrifugation for 10 min at 18.0× *g*, 50 µL of the clear supernatant was mixed with 100 µL of water/formic acid/ammonium formate (100:0.1:0.02). Hereafter an aliquot of 2 µL was injected onto the UPLC column.

Tumor tissue samples were directly frozen and stored at T < −80 °C until analysis. Tissues were homogenized in 400 µL of blank human plasma with a tissue-lyser (Qiagen, Hilden, Germany) and a stainless-steel bead (5 mm) for 90 s at 50 Hz. Homogenized tissue samples were further processed as plasma samples as described above.

### 4.9. Statistical Analysis

All statistical analysis and graphs were performed/created using Graphpad Prism software version 9.0.0 (GraphPad Software, San Diego, CA, USA). Significant differences between the area under the curve were evaluated using an unpaired *t*-test. All other significant differences were evaluated using a one-way ANOVA test. *p*-values below 0.05 were considered significant.

## Figures and Tables

**Table 1:** Olaparib levels in tumor and plasma of vehicle/olaparib animals. Animals 9–12 received an oral dose of 100 mg/kg olaparib approximately 2 h before sacrifice. Animal 40 was sacrificed 7 days after the final olaparib administration. ’-‘ indicates values under detection limit.

Animal Number	Olaparib Level in Tumor Tissue (ng/mg)	Olaparib Level in Plasma (nM)
9	2.17	13,234
10	0.144	-
11	0.747	2864
12	1.35	8047
40	-	-

## Data Availability

Not applicable.

## Supplementary Materials

The following supporting information can be downloaded at: https://www.mdpi.com/article/10.3390/ijms23148037/s1.
