# Supplementary material for: Preclinical Assessment of the Combination of PSMA-Targeting Radionuclide Therapy with PARP Inhibitors for Prostate Cancer Treatment
Source: Int J Mol Sci. 2022 Jul 21;23(14):8037. doi: 10.3390/ijms23148037 (PMC9316488; doi:10.3390/ijms23148037)
Supplement: Supplementary file 1 [file ijms-23-08037-s001.zip › ijms-1798880-supplementary.pdf]

International Journal of
Molecular Sciences

MDPI

Article

Preclinical Assessment of the Combination of PSMA-Targeting Radionuclide Therapy with PARP Inhibitors for Prostate Cancer Treatment

Eline A. M. Ruigrok [1,2], Nicole S. Verkaik [3], Erik de Blois [1], Corrina de Ridder [1,2], Debra Stuurman [1,2], Stefan J. Roobol [1,3], Dik C. Van Gent [3], Marion de Jong [1,†], Wytske M. Van Weerden [2] and Julie Nonnekens [1,3,*]

Supplemental figures

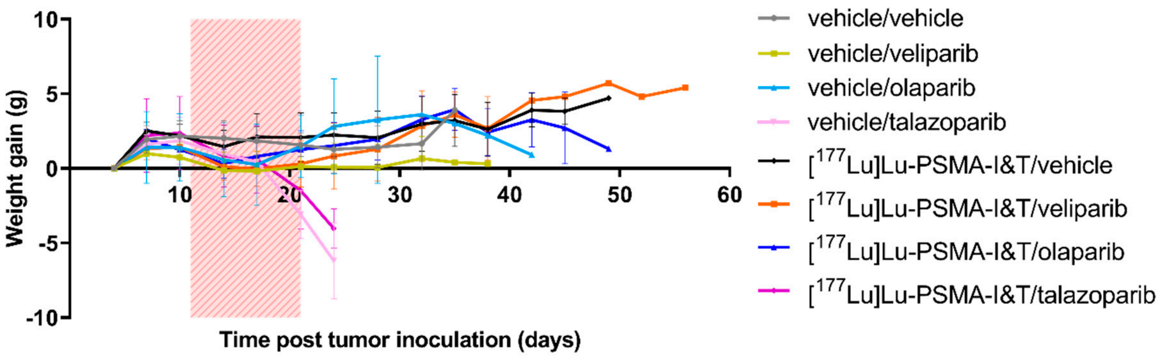

Figure S1. Weight gain over time of mice from the *in vivo* study (see Figure 3).

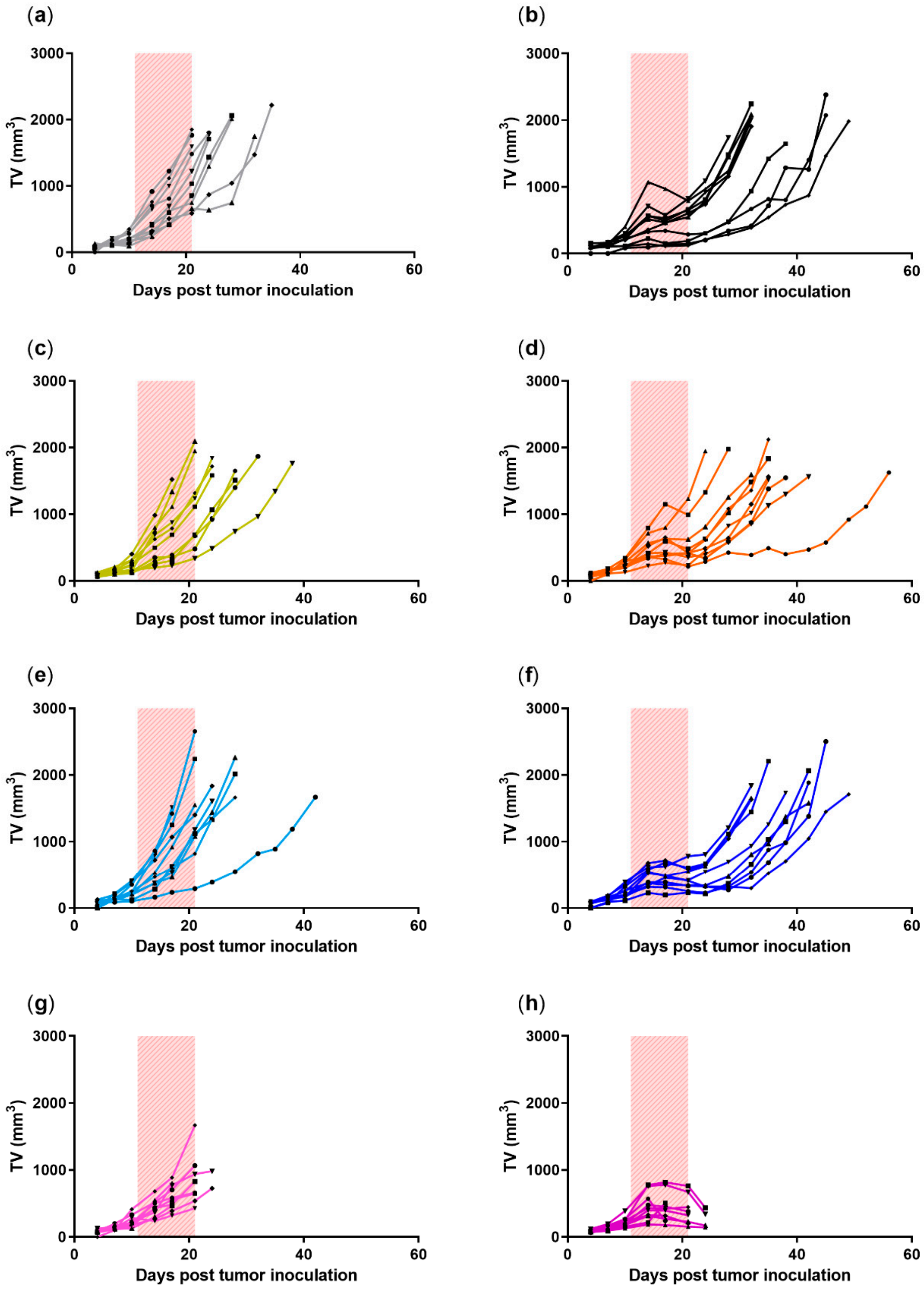

Figure S2. Tumor growth of individual mice in separate graphs per group. (**a**) Group1 vehicle/vehicle, (**b**) group 2 [177Lu]Lu-PSMA-I&T/vehicle, (**c**) group 3 vehicle/veliparib, (**d**) group 4 [177Lu]Lu-PSMA-I&T/veliparib,(**e**) vehicle/olaparib, (**f**) [177Lu]Lu-PSMA-I&T/olaparib, (**g**) vehicle/talazoparib and (**h**) group 8 [177Lu]Lu-PSMA-I&T/talazoparib. The red box indicates the treatment period with one injection of [177Lu]Lu-PSMA-I&T or vehicle at day 11 and subsequent daily dosing by oral gavage of the PARPi or vehicle from day 11 until day 21.
